# A blended design in acute care training: similar learning results, less training costs compared with a traditional format

**DOI:** 10.1007/s40037-014-0109-0

**Published:** 2014-01-30

**Authors:** Mary E. W. Dankbaar, Diana J. Storm, Irene C. Teeuwen, Stephanie C. E. Schuit

**Affiliations:** 1 Desiderius School, Erasmus Medical Center Rotterdam, Room Gk 657, PO Box 2040, 3000 CA Rotterdam, Burg. S’ Jacobplein 51, 3015 CA Rotterdam, the Netherlands; 2 Training Center for Health Professionals, Erasmus Medical Center Rotterdam, Rotterdam, the Netherlands; 3 Department of Internal Medicine, Erasmus Medical Centre, Rotterdam, the Netherlands

**Keywords:** Blended learning, Online learning, Learning results, Effectiveness, Acute care education, Training costs

## Abstract

*Introduction* There is a demand for more attractive and efficient training programmes in postgraduate health care training. This retrospective study aims to show the effectiveness of a blended versus traditional face-to-face training design. For nurses in postgraduate Acute and Intensive Care training, the effectiveness of a blended course design was compared with a traditional design. *Methods* In a first pilot study 57 students took a traditional course (2-h lecture and 2-h workshop) and 46 students took a blended course (2-h lecture and 2-h online self-study material). Test results were compared for both groups. After positive results in the pilot study, the design was replicated for the complete programme in Acute and Intensive Care. Now 16 students followed the traditional programme (11 days face-to-face education) and 31 students did the blended programme (7 days face-to-face and 40 h online self-study). An evaluation was done after the pilot and course costs were calculated. *Results* Results show that the traditional and blended groups were similar regarding the main characteristics and did not differ in learning results for both the pilot and the complete programme. Student evaluations of both designs were positive; however, the blended group were more confident that they had achieved the learning objectives. Training costs were reduced substantially. *Conclusion* The blended training design offers an effective and attractive training solution, leading to a significant reduction in costs.

## Introduction

During the last decades, a large number of studies have been performed on the effectiveness of online learning, or technology-enhanced learning. From these studies with students and professionals, both inside and outside the medical domain, the conclusion can be drawn that online learning is at least as effective as more traditional forms of learning, for knowledge and skills and students are equally satisfied with both forms [1–5]. In some studies the combination of online and face-to-face learning (blended learning) has been found to be more effective than traditional learning alone [2, 4, 6] while other studies have shown the same results [7]. There is, however, still little research on the optimal mix of online and instructor-led learning [8]. In this article, we will describe a retrospective effectiveness study on the blended and the face-to-face design of a programme in Acute and Intensive Care for nurses. We will describe the methods we used to analyze its effectiveness and the learning and evaluation results. We will end with a discussion on the cost-effectiveness and implications for health care practice.

The training centre for health professionals of the Erasmus University Medical Center provides postgraduate, registered nurse education programmes for Intensive, Emergency and Cardiac Care. The basic education course in Acute and Intensive Care forms an important baseline for all specialized postgraduate nurse training programmes in Intensive, Emergency and Cardiac Care. These continuous education programmes take 6–18 months and are taken by nurses in combination with their work in hospitals. The programme in Acute and Intensive Care consists of three parts: a respiration, circulation and central nervous system course. The respiration course includes a section on acid–base balance. The students generally experience this specific part of the programme as difficult and were having problems in applying the principles in practice. We looked for improvements in the quality and efficiency of this programme, by applying a blended learning concept, using design features such as practice cases, multimedia, feedback and repetition, which have proven to be effective in online learning [9, 10]. This online study material was offered through a permanently accessible Learning Management System (It’s Learning). The students can decide when to study, (re)do the exercises and whether to collaborate with colleagues or do them alone.

This new design was first launched as a pilot, in order to be able to evaluate the results before implementing it for the rest of the programme. After analyzing the effectiveness of and student reactions to the new course design, the other components of the programme in Acute and Intensive Care were also redesigned to a blended concept. The length of the face-to-face part of the programme was reduced from 11 to 7 days; an online component was added which took ±40 h of self-study.

If this new, blended course design is effective and appreciated by students, an improvement in course quality and efficiency in training time (and thus cost reduction) can be realized.

In this time of reduced budgets and growing demands on knowledge and skills of health care professionals, this is an important asset for health care organizations. For students, blended learning not only offers the opportunity of flexible, ‘anytime, anywhere’ learning, adaptable to work pressure and personal conditions. It also offers the opportunity to personalize learning: specific, complex parts of the content can be exercised as often as desired, until they are profoundly understood and can be applied in practice, without risks for patient care [3]. Although there are a large number of articles on online learning, evaluation studies remain less common [11]. Comparing two instructional methods or formats in terms of learning outcomes is relevant to bring the field of online learning further [12].

## Methods

### Traditional and blended design of pilot programme

The traditional design of the pilot programme (‘acid–base balance’, part of the course on respiration) consists of a 2-h face-to-face (f2f) lecture and a 2-h f2f workshop (practice exercises on acid–base balance, blood gas assessment, etc.). The new ‘blended’ design of this programme consists of the same 2-h lecture; the workshop is replaced by 2-h online self-study material. The material includes: short web lectures (explaining essentials), a range of exercises and examples with feedback.

### Participants of the pilot

The test results of the students (*n* = 57) of the traditional ‘acid base’ part of the spring 2011 programme were compared with the test results of the blended design group in autumn 2011 (*n* = 46). In order to evaluate the appreciation by students, we used a survey which was sent to both groups after the course.

### Traditional and blended design of programme on Acute and Intensive Care

After analysis of the results and the conclusion that the new approach turned out to be successful, the blended design was applied to the rest of the programme on Acute and Intensive Care for all students.

The three main parts—respiration, circulation and central nervous system—were redesigned, reducing the number of face-to-face training days from 11 to 7, spread over 2 weeks. The seven training days mainly consisted of lectures, explaining the course material. The (original) 4 days of workshops were replaced by online study material with the same format as in the pilot (short web lectures, examples and exercises with feedback). Several exercises now had a game format. This takes about 40 h of self-study and can be used to prepare for the lectures and for self-study.

### Participants of the programme on Acute and Intensive Care

Students from the basic programme on Acute and Intensive Care in January 2012 followed a traditional design programme (*n* = 13–16, depending on the specific test on respiration, circulation or the nervous system) and students from the same programme in May 2012 followed a blended design (*n* = 27–31, depending on the test). All students have experience as a nurse and are currently working as trainees on a special care unit. They all started with the basic programme on Acute and Intensive Care and continued with a specialized training programme. In Table 1 the characteristics of the two groups are compared.

### Knowledge tests

The knowledge test on ‘acid–base balance’ includes 15 questions, as part of 60 multiple choice questions on respiration (2–4 alternatives). It is part of a summative exam in which the pass/fail cut-off is defined in a test matrix beforehand. The 15 test questions from both pilot groups are drawn from the same question pool, in line with the test matrix. They are equivalent in difficulty and in the number of alternatives.

The Acute and Intensive Care programme has three separate knowledge tests, in time sequence: on the central nervous system (45 questions), on circulation (53 questions) and on respiration (60 questions). The tests are summative exams and similar to the pilot test (multiple choice answers with 2–4 alternatives, pass/fail cut-off defined beforehand, questions are equivalent in difficulty and in the number of alternatives). All tests were taken at the end of each programme part. The test results of participants of the traditional (group 1) and blended group (group 2) of the pilot (2011) were compared and, a year later, the test results of the participants of the complete programme (2012) were again compared for group 1 and 2.

### Evaluation

A short evaluation was done after the pilot programme; the survey was sent to the participants, including a number of statements (4-point scale) and open questions.

### Statistics

We did a reliability analysis of the knowledge tests (Cronbach’s alpha), T-tests to compare means of the knowledge test results and a Mann–Whitney test to compare the evaluation results, using SPSS version 20.

### Calculation of training costs

We calculated the training costs for a hospital to have employees follow the traditional or blended design, by comparing the direct and indirect costs, assuming: (a) self-study time is not paid; (b) travel costs are €20—per day on average; (c) indirect costs (costs for the absence of an employee) for nurses are €50—per hour on average, €400—per day.

## Results

### Comparability of groups

In Table 1 the characteristics of the participants in the pilot group and the complete programme on Acute and Intensive Care are described for group 1 (traditional) and 2 (blended).

Although students in the traditional pilot group [1] were slightly younger and more often followed higher education compared with the blended pilot group [2], there were no significant differences between the two groups for the pilot or complete course.

**Table 1:** Characteristics of participants in the traditional and blended groups, for the pilot programme and the complete course

	Pilot group 1	Pilot group 2	*P* value	Course group 1	Course group 2	*P* value
	Traditional	Blended		Traditional	Blended	
	2011 (*n* = 57)	2011 (*n* = 46)		2012 (*n* = 16)	2012 (*n* = 31)	
Age			0.92			0.99
Age <30	40 (70 %)	29 (63 %)		9 (56 %)	17 (55 %)	
Age ≥30		17 (37 %)		7 (44 %)	14 (45 %)	
		17 (30 %)				
Sex			0.93			0.62
Female	46 (80 %)	39 (85 %)		8 (50 %)	24 (77 %)	
Male	11 (20 %)	7 (15 %)		8 (50 %)	7 (23 %)	
Previous education			0.88			0.86
Higher education	28 (50 %)	18 (39 %)		7 (44 %)	10 (32 %)	
Intermediate education	29 (50 %)	28 (61 %)		9 (56 %)	21 (68 %)	
Type of hospital			0.89			0.95
University hospital	21 (36 %)	21 (46 %)		6 (38 %)	13 (42 %)	
General hospital	36 (64 %)	25 (54 %)		10 (62 %)	18 (58 %)	
Specialization			0.99			0.99
Intensive care	18 (32 %)	8 (17 %)		6 (37.5 %)	5 (16 %)	
Emergency care	10 (18 %)	7 (15 %)		4 (25 %)	10 (32 %)	
Cardiac care	6 (10 %)	6 (13 %)		0	1 (3 %)	
Others	23 (40 %)	25 (54 %)		6 (37.5 %)	15 (48 %)	

### Reliability of knowledge tests

The reliability (Cronbach’s alpha) for the different test versions on ‘acid–base balance’ was between 0.37 and 0.67 (for group 1 the average α of the test versions was 0.57; for group 2 the average α was 0.47). This reliability is relatively poor, probably because of the small number of questions [15].

The Cronbach’s alpha for the different test versions of the tests on respiration, circulation and the central nervous system was between 0.35 and 0.77 (the average α of the test versions for group 1 was 0.63, for group 2 it was 0.65); this reliability is moderate. The tests on circulation for both groups had a good reliability (0.77 and 0.68).

### Pilot group: test results

Group 1 and 2 both answered 76 % questions about the acid–base balance correctly: the blended group had the same results on the knowledge test as the traditional group.

### Pilot group: evaluation of the new design

The participants of the traditional and blended group evaluated the pilot programme roughly equally positively, except for the statement ‘I have achieved the course objectives’. The blended group was more self-confident in this statement (U = 390, *P* = 0.023 Table 2).

**Table 2:** Opinions of students on the traditional and blended programme, ‘acid–base balance’ pilot

Question	Students group 1 (traditional, *n* = 22)	Students group 2 (blended, *n* = 31)	Mann–Whitney scores (U and *P* value)
I have achieved the course objectives			
Not at all	0 %	0 %	U = 390 *P* = 0.023
Somewhat	17 %	3 %	
Reasonably well	22 %	13 %	
Very well	50 %	82 %	
No answer	11 %	2 %	
This education format suits my learning style			
Not at all	0 %	0 %	U = 393 *P* = 0.100
Somewhat	0 %	0 %	
Reasonably well	67 %	41 %	
Very well	33 %	53 %	
No answer	–	6 %	
I can now understand the clinical characteristics of a patient from the blood gas analyses			
Not at all	0 %	3 %	U = 364 *P* = 0.477
Somewhat	40 %	9 %	
Reasonably well	33 %	73 %	
Very well	27 %	12 %	
No answer	–	3 %	

Comments on open questions from students were: ‘The explanation of the teacher during the workshop was good’ (group 1) and ‘It’s pleasant to be able to practise independently’, ‘frequently repeating the exercises is useful’ (group 2).

### Basic Acute and Intensive Care programme: test results

**Fig. 1:**
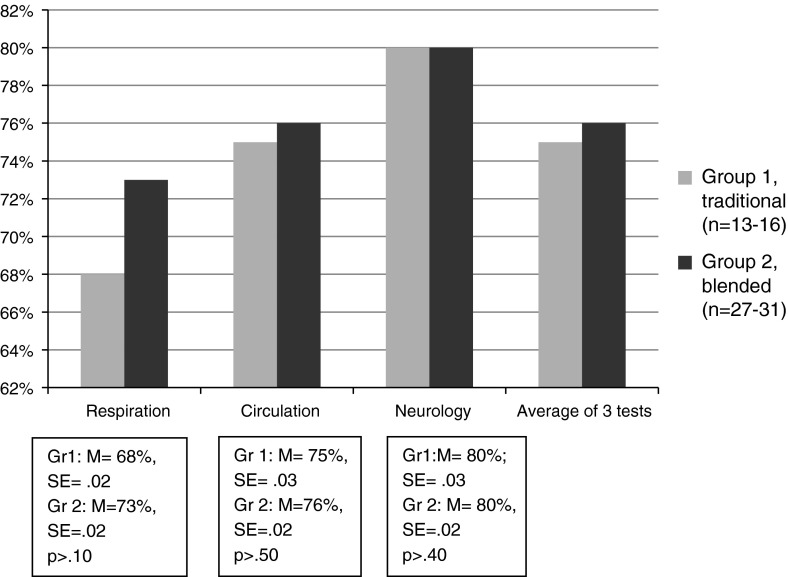
Results of the knowledge test for the traditional and blended groups for the complete Acute and Intensive Care programme (% correct answers)

As Fig. 1 shows, the test results of the blended group [2] on the respiration test were slightly better compared with group 1 although not significant (*P* > 0.10). The respiration test (60 items) contains more abstract content compared with the other tests. The results on the circulation (53 items) and neurology (45 items) tests were similar for both groups. Both groups scored better on the neurology test, probably because of the smaller number of items.

### Training costs

The costs of training health personnel include direct training costs (course fee and travel expenses) and indirect costs (costs for the absence of an employee). The blended Acute and Intensive Care course has been reduced in price by the Erasmus MC training centre from € 1,350 to € 1,270 compared with the traditional format. As contact time is reduced by 4 days, travel costs are reduced by € 80 per employee on average. Direct training costs for a health organization are therefore reduced to € 160 per employee. Absence from work is reduced from 11 to 7 days, for 32 h the indirect cost saving is € 1,600 for nurses on average.

For hospitals that send their postgraduate nurses to training, direct and indirect costs of the training can be reduced by € 1,760 per person (€ 5,970 for the traditional model to € 4,210 for the blended model) by using this blended training model for the course on Acute and Intensive Care.

## Discussion

We found similar learning results for a blended course design (one-third less contact time and more self-study) compared with a traditional design (more contact time and less self-study). Looking at the background characteristics of the two groups, a higher score for the traditional group was to be expected, because they are younger and more highly educated. Reducing the face-to-face (f2f) training time by one-third (from 11 to 7 days in 2 weeks’ time) and adding 40 h of online education led to equally effective learning. Participants were satisfied with the more interactive way of learning (although they needed to spend more free time studying) and were more confident that they had achieved the goals of the course. On the respiratory test (experienced as the most ‘difficult’ one), they performed somewhat, but not significantly better (although the number of participants was small). Particularly with complex subjects, the blended design offers the possibility for customized, frequent exercise.

Although we did not randomize the two student groups and cannot be sure what their knowledge levels were before the training, we were able to confirm that they are comparable on a number of important characteristics. These findings were also confirmed in other small-scale studies [13]. Further research with larger groups is necessary to validate these outcomes, preferably randomized studies with a posttest-only design [14]. One study with a randomized trial on traditional lecturing versus *additional* online learning (blended) reported higher levels of newborn examination skills in the blended learning group [15]; however, this may be caused by a longer learning time. In another randomized trial learning time was fixed, and the same results for the f2f and blended design were reported [16]. A limitation of this study is the fact we do not have data on the learning time (the response from students was too low to make reliable conclusions). Very few studies report on learning time in blended design studies. One qualitative study reported that students welcome the flexibility, but some feel the online component is invasive in their everyday life, specifically following a day at work [17].

In addition, it would be interesting to find out more about the most optimal mix and conditions for implementation of a blended design. The group in this research was motivated, as they work in a clinical setting where knowledge on acute care subjects is essential. For other groups who are not yet working, the results might be different and another blended mix might be advisable.

As the total training costs are significantly reduced by 30 %, this training design is an attractive model for health organizations that want to offer efficient and cost-effective training. For the delivering organization, redesigning a course requires both time and expertise in the development of effective and attractive e-learning material. Although the initial investment is high, the investment is worth the costs for training institutions with a large number of participants, because teacher costs are also reduced. In addition, it stimulates evaluation of the didactic quality of the course. The Erasmus University MC training centre has decided to implement the blended design for all specialized training programmes for nurses.

## Conclusions

For the basic Acute and Intensive Care programme at Erasmus MC we have shown that when a course is redesigned from 11 days face-to-face training (in 2 weeks) to a blended design of 7 days f2f training, complemented by online self-study material, learning outcomes remain the same. Students are satisfied with this new design, as they can tailor learning to their own needs and it offers flexibility. For the affiliated hospitals it is important that the training costs are reduced and nurses are more available for patient care.

## Essentials

- Blended learning, with one-third less face-to-face training time and more online self-study is equally effective in learning outcomes for a postgraduate course on Acute and Intensive Care.
- Highly interactive online self-study material enables students to study complex subjects in a customized and flexible way, being able to exercise as frequently as they wish.
- Students are satisfied with the new blended design, although it implies more self-study time.
- For health organizations a blended design leads to a significant reduction of costs, mainly because of savings in indirect training costs. Health professionals are more available for patient care.
- More research is needed with larger and different groups of participants and courses to learn more about the optimal blended mix and implementation conditions.
